# Contrast Sensitivity Is a Significant Predictor of Performance in Rifle Shooting for Athletes With Vision Impairment

**DOI:** 10.3389/fpsyg.2018.00950

**Published:** 2018-06-26

**Authors:** Peter M. Allen, Rianne H. J. C. Ravensbergen, Keziah Latham, Amy Rose, Joy Myint, David L. Mann

**Affiliations:** ^1^Department of Vision and Hearing Sciences, Anglia Ruskin University, Cambridge, United Kingdom; ^2^Vision and Eye Research Unit, Anglia Ruskin University, Cambridge, United Kingdom; ^3^Department of Human Movement Sciences, IPC Research and Development Centre for the Classification of Athletes with Vision Impairment, Amsterdam Movement Sciences and Institute of Brain and Behavior Amsterdam, Vrije Universiteit Amsterdam, Amsterdam, Netherlands; ^4^Life and Medical Sciences, University of Hertfordshire, Hatfield, United Kingdom

**Keywords:** paralympic sport, vision impairment, shooting, contrast sensitivity, visual acuity

## Abstract

**Purpose:** In order to develop an evidence-based, sport-specific minimum impairment criteria (MIC) for the sport of vision-impaired (VI) shooting, this study aimed to determine the relative influence of losses in visual acuity (VA) and contrast sensitivity (CS) on shooting performance. Presently, VA but not CS is used to determine eligibility to compete in VI shooting.

**Methods:** Elite able-sighted athletes (*n* = 27) shot under standard conditions with their habitual vision, and with their vision impaired by the use of simulation spectacles (filters which reduce both VA and CS) and refractive blur (lenses which reduce VA with less effect on CS). Habitual shooting scores were used to establish a cut-off in order to determine when shooting performance was ‘below expected’ in the presence of vision impairment. Logistic regression and decision tree analyses were then used to assess the relationship between visual function and shooting performance.

**Results:** Mild reductions in VA and/or CS did not alter shooting performance, with greater reductions required for shooting performance to fall below habitual levels (below 87% of normalized performance). Stepwise logistic regression selected CS as the most significant predictor of shooting performance, with VA subsequently improving the validity of the model. In an unconstrained decision tree analysis, CS was selected as the sole criterion (80%) for predicting ‘below expected’ shooting score.

**Conclusion:** Shooting performance is better predicted by losses in CS than by VA. Given that it is not presently tested during classification, the results suggest that CS is an important measure to include in testing for the classification of vision impairment for athletes competing in VI shooting.

## Introduction

Classification is a process in which athletes with impairment are tested to determine whether they are eligible to compete in Paralympic sport events, and if so, which ‘class’ they should compete in. Classification aims to ensure that athletes compete against others who have a similar level of impairment (International Paralympic Committee, 2007, 2014). The Classification Code of the International Paralympic Committee (IPC) explicitly details the need for each sport to develop and implement their own sport-specific evidence-based system of classification (Tweedy and Vanlandewijck, 2011; Tweedy et al., 2014). Therefore, the system should be developed on the basis of scientific evidence which demonstrates the relationship between the degree of impairment and performance in that sport. Although this process has for some time been underway for athletes with physical or intellectual impairments, at this stage there has been minimal change to the classification systems for athletes with vision impairment (VI) (Ravensbergen et al., 2016).

Shooting is a sport that is particularly attractive to athletes with vision impairment because, in the VI-adapted form of the sport, competitors can rely on sound rather than (or in addition to) vision to guide the direction of the gun barrel toward the target. The air rifle is fitted with an acoustic mechanism that allows the athlete to ‘sight’ via an audio signal: the closer the gun barrel is directed toward the center of the target, the higher will be the pitch of the tone. This aiming mechanism is mounted on the air rifle, with the athlete able to listen to the signal through headphones. Shots are fired and the score is measured opto-electronically. These adaptations to the sport make it very accessible and attractive to persons with high levels of vision impairment. Myint et al. (2016) have recently shown there to be no association between the level of vision impairment and shooting performance with auditory guidance in a group of athletes with VI [with visual acuity (VA) worse than or equal to 1.0 logMAR units]. That is, athletes who were completely blind, or had severe vision impairment, were able to perform just as well as those with much less vision impairment. These findings suggest that athletes are successfully able to use the auditory information to compensate for their impairment in VI rifle shooting independent of their level of VA.

The current classification system for VI-shooting requires athletes to be assessed using up to two different tests of visual function: distance VA and/or visual field, with eligible athletes classified to compete in one of the B3, B2, or B1 classes (from lowest to highest impairment) depending on their level of impairment (see **Table 1**). These classes were created some time ago and were based on the World Health Organization’s criteria for low vision and blindness (World Health Organization, 2004). Therefore, the present system is not evidence-based or sport specific, and so does not meet the criteria for classification set out by the IPC.

**Table 1:** The criteria for the three sports classes for athletes with vision impairment.

Class	Criteria
B3	VA is between 1.0 and 1.5 logMAR and/or the VF is constricted to a radius of less than 20 degrees
B2	VA is between 1.5 and 2.6 logMAR and/or the VF is constricted to a radius of less than 5 degrees
B1	VA is less than 2.6 logMAR

*VA, visual acuity; VF, visual field.*

The current system of classification measures only the VA and visual field, however, there are other aspects of vision that could be related to performance yet are not presently accounted for during classification (Ravensbergen et al., 2016; Mann and Ravensbergen, 2018). For instance, decreases in contrast sensitivity (CS) have been shown to be associated with an increased risk of falls in the elderly (Lord et al., 1991) and for poorer performance when driving (Wood, 2002). Allen et al. (2016) recently showed that moderate reductions in both VA and CS, when in conjunction with each other (poorer than 0.5 logMAR and 0.8 logCS, respectively), were associated with significantly poorer shooting performance in the unadapted form of the sport (without auditory guidance). Allen et al.’s (2016) findings helped to demonstrate the degree of impairment that would be required to result in a decrease in shooting performance. However, in that study, VA and CS co-varied strongly, meaning that any loss in VA was associated with a commensurate loss in CS. Accordingly, it was not possible to set *separate* MIC for VA and CS. Independent criteria are necessary because some medical conditions can selectively impair VA more than CS, and vice versa (Elliott, 2006). It remains important to determine the relative influence of losses in VA and CS in order to set an evidence-based MIC for VI shooting.

An evidence-based MIC should ensure that athletes who are disadvantaged as a result of their impairment in the *unadapted* form of the sport are eligible to compete in the *adapted* form of the sport (Mann and Ravensbergen, 2018). The reason why the MIC should be based on performance in the unadapted rather than the adapted form of the sport is that para-sports should cater for athletes whose performance would be significantly impacted by impairment, without the assistance of any adaptations such as a guide or auditory guidance. Doing so would ensure that athletes compete only against others who have an impairment that impacts performance. If the MIC were to be established on the basis of the *adapted* form of the sport, then it would be done with the adapted rules in place (e.g., with a guide, blindfold, or auditory guidance). If the *auditory* guidance used in VI shooting proved to be as effective as *visual* guidance, then a completely blind athlete would be able to compete without disadvantage against a fully sighted opponent. Therefore, there would be no level of vision impairment that decreased performance in the sport, making it impossible to define a MIC for VI shooting.

The aim of this study was to determine the extent to which an impairment to VA and CS would impact performance in shooting. The shooting performance of international-level shooters without vision impairment was assessed in the *unadapted* form of the sport while wearing simulation spectacles (sim-specs) that simultaneously reduced VA and CS, and refractive lenses that primarily reduced VA. The results were expected to demonstrate the independent levels of VA and CS that would decrease performance in competitive shooters, and in the process, would provide guidance for an appropriate MIC to be used for Paralympic shooting for athletes with vision impairment.

## Materials and Methods

### Participants

Twenty-seven elite able-sighted shooters (14 male, all competing at international level at the time of testing) took part in the study (M_age_ ± SD = 26.9 ± 12.6; range 17–56 years). Participation in the study was voluntary, with all athletes agreeing to participate without reward or incentive. The Faculty Research Ethics Panel at Anglia Ruskin University, Cambridge, United Kingdom, gave ethical approval for the study. All participants provided written informed consent and the research was conducted in accordance with the tenets of the Declaration of Helsinki.

### Procedure

All data were collected during training camps or competitions at the West Midlands Regional Shooting Centre or the Sport Wales National Centre. The shooting ranges were equivalent, both being 10 m indoor rifle ranges with standardized lighting at the target of a minimum of 1500 lux and a maximum of 1800 lux. Vision and shooting performance were assessed in each of the different vision conditions.

### Measurement of Vision

For each vision condition, two tests of visual function were performed viewing with the shooting eye under standardized lighting conditions (measured as ≈200 lux or ≈32–64 cd/m^2^). First, a test of *distance visual acuity* was performed, given that it was the test used for classification at the time of testing. VA was measured using an externally illuminated ETDRS LogMAR letter chart at 4 m (2000 Series Revised, Precision Vision, La Salle, IL, United States). Letter-by-letter scoring was used with the acuity measured in logMAR units. On the logMAR scale, lower logMAR scores indicate better VA. Although a tumbling-E logMAR chart is used currently for the purposes of classification, the ETDRS logMAR chart produces very similar levels of acuity (Bourne et al., 2003). Second, a test of *contrast sensitivity* was performed using a Pelli-Robson chart (Pelli et al., 1988) at 1 m. A letter-by-letter scoring method was used. A Pelli-Robson chart was used because it is widely considered to be the gold-standard test for CS.

### Simulated Vision Impairment

#### Part 1

Nineteen participants completed part one of the studies. Cambridge sim-specs were used to simulate increasing levels of vision impairment (Goodman-Deane et al., 2013). The sim-specs consist of diffusing filters that block and scatter light to reduce visual performance and are mounted in cardboard frames so that both eyes look through separate filters. The filters can be used individually, or with several in combination to provide progressive increases in simulated impairment. In six separate conditions, we used one through to six filters in front of both eyes to simulate six different levels of vision impairment (termed ‘Level 1’ to ‘Level 6’). The non-shooting eye was also occluded as is normal practice in shooting. The sim-specs result in decreases in both VA and CS (Rae et al., 2015), and so both measures were assessed in each level of simulated impairment to examine the combined effect of decreases in VA and CS on shooting performance. In order to investigate the independent effects of VA and CS, vision was also impaired in three additional conditions using +1.00D, +2.00D, and +3.00D spherical trial lenses placed in front of the participant’s shooting eye. This latter simulation was expected to reduce VA much more than it would reduce CS. These data have previously been presented (Allen et al., 2016) using different analyses to those employed here.

#### Part 2

After initial data analysis, further data were collected on an additional eight participants in order to supplement data around the key regions where performance decreased, as identified in Allen et al. (2016). These data have not previously been presented. We used one to four filters in front of the shooting eye to simulate four different levels of vision impairment (termed ‘Level 1’ to ‘Level 4’). The Levels 5 and 6 filters were omitted because they consistently resulted in a large decrease in shooting performance, using a level of impairment that was clearly beyond that necessary to decrease performance. Vision was also impaired using +1.50D, +2.00D, and +2.50D refractive lenses placed in front of the participants’ shooting eye. These powers were selected in order to maximize the observations around the level of vision that resulted in a reduction in shooting performance.

### Effect of Simulated Vision Impairment on Shooting Performance

In both parts of the study participants stood while shooting a regulation competition air rifle toward a regulation high contrast target placed at the end of a 10 m shooting range. Scoring was performed using an electronic scoring system (SCATT) rather than through the use of actual pellets. The target replicated that used during competition, consisting of ten rings so that there was a central circle surrounded by nine concentric annuli, with the athlete scoring 10 for a hit in the central circle, nine for the immediately surrounding annulus, eight for the next annulus, and so on. Although any further subdivisions were not visible to the participants, the SCATT scoring system further subdivided each of the ten rings into 10 concentric annular ‘score zones’ of equal width with increments of 0.1 between zones. As a result, the highest score for an individual shot was 10.9.

Participants first took part in a 5-min warm up without any additional lenses or filters. Subsequently, in each vision condition, three shots were taken toward the target without any additional practice trials. The scores for the three shots were used for the analysis of performance. Performance feedback was available on a 13-inch screen placed approximately 1 m from the participant, as is normally the case when in competition. We chose to include only a small number of shots in each condition because this is more representative of the demands of competition, where international athletes must perform at a high level on every shot. Participants first shot with their habitual vision (no additional lenses or sim-specs in place) before shooting in each of the nine (Part 1) or seven (Part 2) simulated impairment conditions that were presented in a randomized order for each participant.

### Statistical Analysis

Data from parts 1 and 2 were combined to investigate the individual and combined effects of impairments to VA and CS on shooting performance. Data collected when shooting with habitual vision were used to determine the expected score for each individual.

Scores of shooting performance in the presence of simulated vision impairment were normalized as a percentage of the participant’s performance with habitual vision. To calculate this, we normalized each individual’s shot by that person’s mean across their three shots in the habitual condition (i.e., $\mathrm{Normalised}\;\mathrm{shot}\;score=\;\frac{\mathrm{Individual}\;\mathrm{shot}\;\mathrm{score}}{\mathrm{Three}\;\mathrm{shot}\;\mathrm{average}}*100)$). Shooting performance, even in the habitual vision condition, will naturally vary as a result of chance. In order to establish a boundary for ‘normal’ or expected shooting performance, each individual’s shots in the habitual condition were first normalized according to the average of their three shots. All shots across all participants in the habitual condition were then combined to calculate a 99% confidence interval. The lower boundary of the 99% CI was used as the level below which a shot would be considered to be ‘below expected’ performance. We were interested in the level of vision impairment that would consistently result in shots whose score was ‘below expected.’

In order to determine the specific degree of impairment to VA and CS that would lead to ‘below expected’ shooting performance, two types of analysis were used: logistic regression analysis and decision tree analysis.

### Regression Analysis

Logistic regression was employed, using the forward stepwise selection method minimizing the Akaike information criterion (AIC) (Burnham and Anderson, 2004; Wagenmakers and Farrell, 2004), to determine which factors (VA, CS and/or a VA^∗^CS interaction term) best predicted when a participant would shoot at a level ‘below expected.’ Probability for entry of a stepwise term was 0.05, and probability for exclusion was 0.10. The regression model was developed using a training data set that consisted of 80% of our observations that were randomly selected from the pooled data across participants but balanced for the overall proportion of shooting performance levels. The model was then tested for accuracy on a test data set that consisted of the remaining 20% of observations.

### Decision Tree Analysis

To identify the MIC based on a combination of VA and CS, we used a tree-based classification model (‘tree’ package in R) (Zhang, 2016). The approach used in this method was *recursive binary splitting*, where the best split in the performance data is continuously made using criteria for both VA and CS. The best split in this case is the split that leads to two nodes that are most ‘pure,’ meaning that they optimally separate ‘expected’ or ‘below expected’ performances. After the first split, the same process was continued until the stopping criterion was met, requiring at least five data points in each node. The decision tree was then pruned by determining the optimal level of complexity of the tree using cross-validation. The tree was built and pruned using the same training data set used for the logistic regression, and then tested for accuracy using the same test data set used for the logistic regression, ensuring that any difference in results found between the regression and decision tree analyses could not be explained by differences in the data sets used to train and test the two approaches.

In order to determine whether CS would better discriminate performance when added to the existing measure (VA), we used decision-tree analysis while employing a constrained analysis model. To do so we used the same decision tree analysis, but first entering only VA as a predictor. The first split criterion for VA was then used to create a subset of observations that met this VA criterion that predicted ‘below expected’ shooting performance. As a third and final step, we ran the decision tree analysis again on this subset of observations, now using both VA and CS as input variables to clarify whether CS provided additional information when VA was used as the main predictor for ‘below expected’ shooting performance.

## Results

### Participant Demographics

The mean habitual VA of the 27 athletes was -0.06 ± 0.10 logMAR (range -0.30 [equating to 6/3 or 20/10 vision] to 0.12 logMAR [approx. 6/7.5 or 20/25 vision]). Mean habitual CS was 1.88 ± 0.13 logCS (range 1.55 to 2.00 logCS).

The mean shooting score with habitual vision (average of three shots for each participant) was 9.8 ± 0.4 (range 9.1 to 10.3). The mean of the individual normalized shot scores across all participants was 100.0 ± 5.0% (99% CI from 87 to 113%). Accordingly, the cut-off point below which performance would be classified as ‘below expected’ was set at 87% of the habitual shooting score.

### Effect of Simulated Vision Loss on VA and CS

**FIGURE 1:**
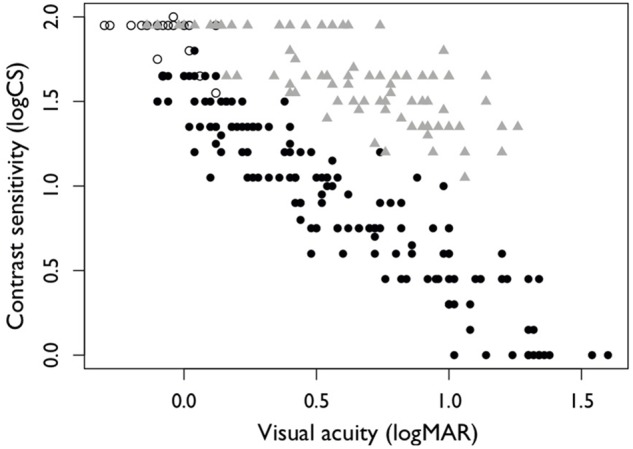
Relationship between visual acuity (VA) and contrast sensitivity (CS) as measured under habitual condition (open circles), with simulated vision impairment by use of sim-specs (filled circles) and by use of refractive blur (gray filled triangles). Mean habitual VA was –0.06 logMAR units, and mean habitual CS was 1.88 logCS units.

As expected, the sim-spec filters reduced both VA and CS whereas the refractive blur lenses primarily reduced VA (**Figure 1**). Specifically, the refractive blur initially affected VA only, and at higher levels reduced CS, but to a lesser extent than when compared to the equivalent VA level using the sim-spec filters. The use of this combined approach allowed for a reasonable separation of VA and CS measures: considering a fixed CS of 1.50 logCS, VA ranged from -0.1 to 1.14 logMAR. Similarly, for a fixed VA of 0.50 logMAR, CS varied from 1.95 (normal) to 0.60 logCS (significant loss).

### Effect of Simulated Vision Loss on Shooting Performance

**FIGURE 2:**
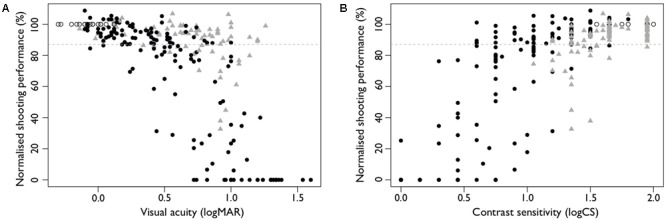
Normalized shooting performance as a function of **(A)** visual acuity and **(B)** contrast sensitivity in the habitual condition (open circles), with simulated vision impairment by use of filters (filled circles) and by use of refractive blur (gray filled triangles). The cut-off below which shooting performance was considered ‘below expected’ is represented by the gray dashed line.

The simulations ensured a wide range of impairment to both VA (-0.14–1.6 logMAR) and CS (1.95 – 0.0 logCS). While mild reductions in VA or CS were not detrimental to shooting performance, greater reductions decreased performance (i.e., normalized shooting performance below 87%, **Figure 2**).

### Regression Analysis

The forward stepwise logistic regression analysis first selected CS into the model, with poorer CS associated with an increased probability of scoring below expected shooting performance (**Table 2**; James et al., 2013).

**Table 2:** Stepwise logistic regression examining the ability of contrast sensitivity (CS) and visual acuity (VA) to predict ‘below expected’ shooting score.

	β	SE β	Odds ratio	95% CI for odds ratio
Step 1		
Constant	3.42	0.59		
CS	–3.27	0.49	0.04^∗∗∗^	0.01–0.09
Step 2		
Constant	1.14	0.83		
CS	–2.63	0.54	0.07^∗∗∗^	0.02–0.17
VA	2.58	0.66	13.2^∗∗∗^	3.79–52

*SE, standard error; CI, confidence interval. For step 1 R^*2*^ = 0.49 (Nagelkerke). For step 2, R^*2*^ = 0.53 (Nagelkerke). ^∗∗∗^p < 0.0001*.

**FIGURE 3:**
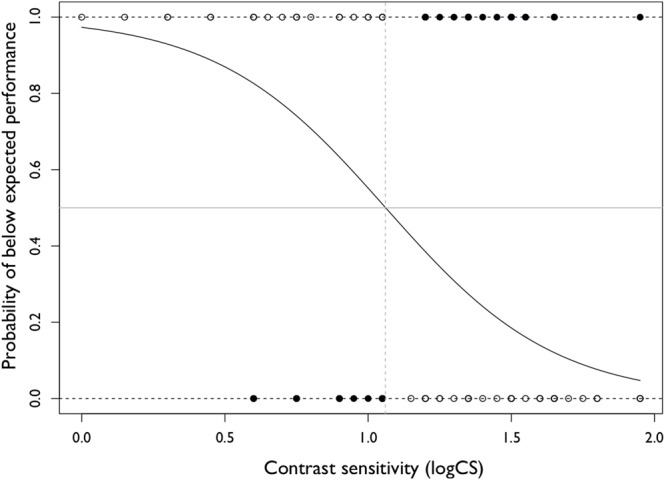
Logistic regression model including CS only. The curve represents the logistic regression model. The horizontal gray line indicates the probability of below expected performance of 0.5 and the vertical dashed gray line indicates the cut off for CS where the probability of below expected performance is 0.5. The open circles indicate observations for which performance is correctly predicted by the CS cut-off and the filled circles indicate observations where performance is incorrectly predicted.

In order to establish a cut-off level of CS below which shooting performance would be considered to be less than normal, we sought to determine the level of CS at which the probability of a below expected shooting score was 50%. On the basis of this, the cut-off for CS using the logistic regression model determined in the first step was 1.05 logCS (**Figure 3**). When testing the accuracy of this cut-off on the test data set, shooting performance was correctly classified in 84% of cases. Correct classification meant that shooting performance was at the expected level when CS was better than the cut-off, and below expected when below the cut-off. The sensitivity for predicting a ‘below expected’ score was 74%, meaning that the CS cut-off was able to correctly identify those with ‘below expected’ scores in 74% of cases. The specificity was 92%, indicating that the CS cut-off was able to correctly identify 92% of the ‘expected’ scores.

The regression model that was produced as a result of the second step of the logistic regression analysis included both CS and VA as significant predictors of shooting performance. Again, a regression equation was established to determine the point below which the probability of less than expected shooting performance was 50% (**Table 2**). When testing this regression model for accuracy on the test data set, the level of shooting performance was correctly predicted in 82% of cases (**Table 3**). The sensitivity for predicting a ‘below expected’ score was 74%, and the specificity was 85%.

**Table 3:** Comparison of the accuracy, sensitivity and specificity of the logistic regression and decision tree models.

	Accuracy	Sensitivity	Specificity
**Logistic regression models**		
Simple model (CS only)	84%	74%	92%
Full model (CS and VA)	82%	74%	85%
CS and VA cut-offs	80%	63%	92%
**Decision tree models**		
Unconstrained	80%	63%	92%
Constrained	72%	53%	88%

*VA, visual acuity; CS, contrast sensitivity*.

A third step in the regression analysis was attempted, but the addition of a variable which expressed the interaction between VA and CS failed to significantly improve the model. Therefore, the impact of CS on shooting performance appears to be relatively independent of that of VA.

In the second step of the regression model, the regression equation could be solved with different combinations of VA and CS. In order to determine a specific cut-off level for VA for which the probability of a below expected shooting score was 50%, we incorporated the CS cut-off defined in the first step, and calculated the VA cut-off (0.63 logMAR). Subsequently, we were able to test our ability to predict an athlete’s level of shooting performance on the basis of set cut-off values for CS and VA (1.05 logCS and 0.63 logMAR, respectively). When applied to the test data set, there was little change in the accuracy of the model. The combination of these VA and CS cut-offs correctly predicted 80% of observations in the test data set (cf. 84% when using CS only). The sensitivity, however, decreased to 63%, while the specificity remained the same at 92%.

This analysis suggests that while impairments to both VA and CS may be independently associated with competitive shooting performance, performance was more strongly influenced by changes in CS. Using the full regression equation, when compared to the simple model which included only CS, the addition of VA did not improve the accuracy of the model. With the use of the simple model which included only CS, we found a reduction in the sensitivity of the model, while specificity remained the same (i.e., more shooters whose performance was affected by vision impairment would be excluded from competition).

### Decision Tree Analysis

#### Unconstrained Model

**FIGURE 4:**
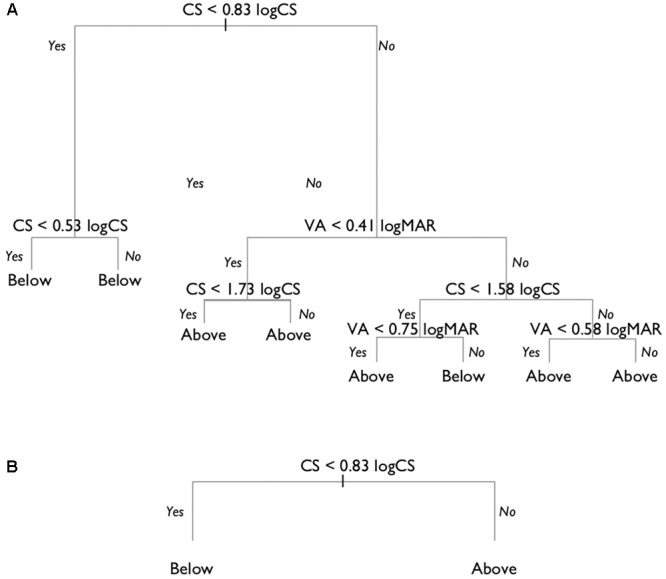
Unconstrained decision tree model. **(A)** The full decision tree prior to pruning. **(B)** The pruned decision tree with two final nodes, showing that shooters with a CS poorer than 0.83 logCS were predicted to have below expected shooting scores, and those with better CS have shooting scores within the expected range.

When a decision tree was built with both VA and CS able to enter the model, the first binary split was made based using a cut-off for CS of 0.83 logCS (**Figure 4A**). This cut-off maximized the differentiation of the expected and below-expected performance scores, respectively, above and below the cut-off value. This process continued until the stopping criterion was met, reaching a maximum of four splits. The process of pruning the decision tree showed that the cross-validation error rate was smallest for a tree with only two terminal nodes (one split; **Figure 4B**).

The performance of the pruned decision tree was tested on the test data set, showing correct classification in 80% of cases. The sensitivity of the model was 63%, and the specificity was 92%. This meant that almost all shooters whose performance was as expected would be correctly classified as ineligible to compete, but that about 37% of the athletes whose performance was below expected would also be classified ineligible to compete. The model shows that CS is the best predictor for below-expected shooting performance, and that VA does not significantly add to the performance of the model.

#### Constrained Model

**FIGURE 5:**
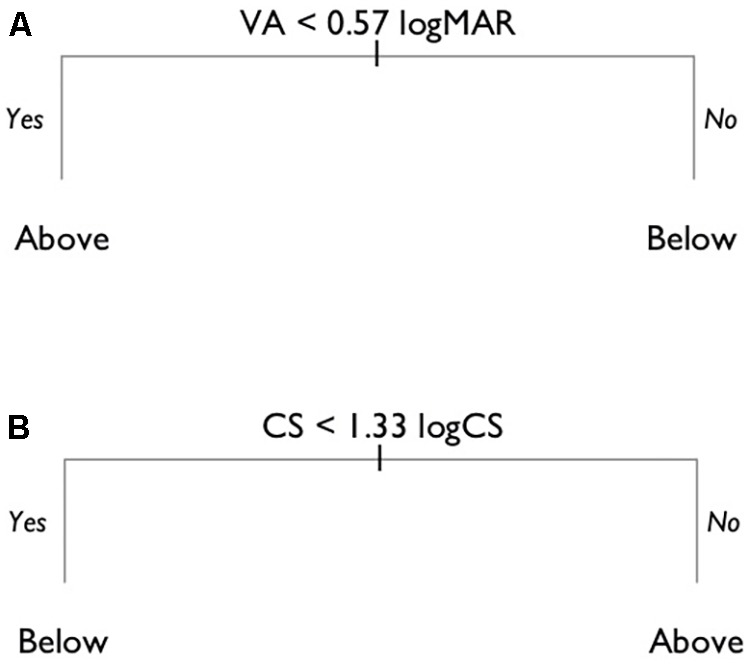
Constrained decision tree model. **(A)** Decision tree model in which VA was first forced into the model, with a VA of 0.57 logMAR found to be the initial cut-off criterion. **(B)** The pruned decision tree on the subset of the data after eliminating the observations where VA was 0.57 logMAR or less.

The unconstrained model indicates that CS alone is the best predictor of below-expected shooting score. However, since VA is the test presently used for classification, and represents the most widely used measure of vision, we wanted to compare the results of the unconstrained model with a model where VA was specifically included. To do so, we used a constrained model in which VA was first entered into the decision tree analysis. This led to a best split for below expected and expected shooting scores at a VA of 0.57 logMAR, where those with worse VA would be expected to have ‘below expected’ performance (**Figure 5A**). In the second step, all observations where the VA was better than 0.57 logMAR (i.e., <0.57 logMAR) were excluded from the data set, and then both VA and CS were entered in the decision tree analysis. Again, the pruning process showed that a model with two final nodes was optimal. This final model showed that CS could be used to predict below-expected shooting performance, even after using a VA of 0.57 logMAR as the initial criterion to predict below expected performance (**Figure 5B**). The model suggests that the shooting performance of those with VA worse than 0.57 logMAR, but CS better than 1.33 logCS, should be unaffected by vision impairment (i.e., ‘expected’ shooting scores).

The model developed in step one (i.e., including only VA), had an overall prediction accuracy on the test data of 62%. The sensitivity was 79%, and the specificity only 50%. Combining the two steps of the model resulted in an overall accuracy of 72%, with a sensitivity of 53%, and a specificity of 88%. This level of performance is slightly less accurate than the unconstrained model.

## Discussion

The aim of this study was to determine the extent to which an impairment to VA and/or CS would impact performance in shooting. Twenty-seven elite able-sighted athletes attempted to shoot toward a target while wearing a series of filters and lenses that simulated different degrees of VA and CS loss. Two different analysis techniques were applied in an attempt to establish the independent impact of losses in VA and CS on shooting performance. The analysis techniques converged to reveal that CS is a better predictor of shooting performance than VA in the unadapted version of the sport, suggesting that it would be a useful addition to tests performed to determine whether a shooter with vision impairment should be eligible to compete in Paralympic competition.

The importance of CS is well established in dynamic tasks (Brown, 1972; Long and Zavod, 2002) and in activities of daily living for individuals with low vision (Elliott et al., 1990). Given the high contrast and static nature of the target used for shooting, it might be considered somewhat surprising that CS would be more predictive of performance than VA. However, the actual task of sighting involves alignment of the shooting eye, the proximal sight, and the distal sight of the air rifle with the target. When the athlete is positioned correctly, they focus on the distal sight on the rifle and align it with an out of focus target positioned 10 m away. The goal of the aiming process is to keep the target centered in the distal sight whilst viewing through the proximal sight (Lujan, 1996). The target is very small, with the center of the target (that scores 10 or above when hit) being 0.5 mm in diameter. As a consequence, the target is blurred and is much more of a low contrast task than might otherwise be expected. It therefore might not be unreasonable to expect CS to be predictive of shooting performance.

### What Should the Cut-Off Be for CS If Used as the Sole Classification Criterion for VI Shooting?

If using CS as the sole criterion for classification, our analyses demonstrate that a value in the region of 0.83–1.05 logCS would be most appropriate. On the basis of the simulated vision impairments, a cut-off in this range would have correctly classified 80–84% of cases (**Table 3**). It is interesting to note that, in low vision clinical practice, a cut-off of 1.05 logCS is used to differentiate between people with ‘noticeable’ and ‘significant’ CS loss, with those having ‘significant’ loss predicted to have difficulty with tasks such as reading (Whittaker and Lovie-Kitchin, 1993; Latham and Tabrett, 2012). This suggests that the visual demands for shooting, at least in terms of CS, are similar to those required for other activities of daily living.

### What Should the Cut-Offs Be If Both CS and VA Are Used as Classification Criteria?

Given the prominence of VA in clinical practice, and that it provides the basis of the current classification criteria, consideration should also be given to the inclusion of VA in the classification criteria. There are two key reasons to continue to consider the inclusion of VA within the criteria. First, from a practical standpoint, there is likely to be resistance from clinicians and classifiers to a system which does not include the measurement of VA, given its prominence as the most widely used measure of vision both in classification and in clinical practice. Second, from a scientific standpoint, we were not able to simulate vision impairment that resulted in a selective loss of CS without also impacting VA. **Figure 1** shows an absence of data points in the lower left-hand corner of the figure (i.e., CS less than 0.85 logCS and VA less than 0.4 logMAR). Accordingly, it remains unknown what might be the impact of poor CS alone on shooting performance (i.e., in conjunction with good VA). It is unlikely that such a visual profile actually exists in persons with vision impairment (Haegerstrom-Portnoy et al., 2000), but if so, it could be that a person with good VA but poor CS might not have a significant activity limitation in shooting as a result of their impairment, and as a result should not be eligible to compete in VI shooting. Therefore, it may be wise to include a MIC for VA to account for these cases.

The results of our analyses suggest that, using our existing data, the addition of VA adds little to the accuracy of the model. Within the logistic regression analysis, VA was selected into the regression at the second step, subsequently explaining an additional 4% of variance in the data. Using the decision tree model, VA was not selected unless the model was constrained by the inclusion of a specific first step requiring the selection of VA. And as is shown in **Table 3**, the inclusion of VA does not improve the accuracy of the models. Therefore, the inclusion of VA would merely act as practical solution to either account for the concerns of clinicians and/or to provide reassurance that athletes with good VA but poor CS would be ineligible to compete. In those cases, further evidence would be required to determine whether those athletes have a significant activity limitation as a result of their impairment.

Our findings suggest that the current clinical tests used during classification (including only VA and VF) may fall short of providing an optimal assessment of functional visual performance relevant to shooting. Indeed, given the utility of CS for predicting performance in other functional tasks (e.g., Lord et al., 1991; Wood, 2002), it continues to be surprising that CS is not measured more regularly in clinical practice, and we suggest that it should be included in the classification of athletes with vision impairment competing in shooting. Moreover, the findings raise the possibility that there could be other measures, such as Vernier acuity and oculomotor control, that could aid in our ability to explain variations in shooting performance.

In the current study, VI was simulated in sighted athletes, rather than testing athletes with vision impairment. This allowed us to access a large number of elite shooters who possessed a standardized level of experience and skill, and to use a repeated measures experimental paradigm that minimized any between-participant variation in shooting performance. A disadvantage of this design is that the sighted athletes had only a short period of time to adapt to the vision loss, particularly when compared to athletes with actual vision loss. Because adaptation may lead to improved performance, the approach that we have used may lead to a slight underestimation of the level of impairment that would decrease performance. Accordingly, if there is, as we have found, a range of levels of impairment that may decrease performance, then there may be reason to lean toward the more severe of the impairments in that range as a suitable MIC to qualify to compete. The simulation approach could be used in conjunction with other approaches, for instance where athletes with vision impairment compete in the unadapted form of the sport, however, this approach is also limited in that athletes with vision impairment are unlikely to be accustomed to shooting *without* auditory guidance.

Performance in all our participants decreased with increasing level of simulated vision impairment. In shooters of similar ability to those who participated in our study the stability of hold has been shown to account for 54% of the variance in shooting score. Other important technical aspects that impact performance include the cleanness of triggering, timing of triggering, postural balance, and aiming accuracy (Era et al., 1996; Mononen et al., 2007; Ihalainen et al., 2016a,b). The simulated vision impairment we induce during this study will no doubt have affected the accuracy of aiming but may also indirectly have affected postural stability. This would be interesting to explore in further work.

## Conclusion

Contrast sensitivity is an important measure to include in a test battery used for the classification of athletes with vision impairment who wish to take part in VI shooting.

## Author Contributions

PA, AR, and JM collected the data. RR, PA, KL, and DM analyzed the data. All authors wrote, reviewed, edited, and approved the manuscript. All authors conceived and designed the study.

## Conflict of Interest Statement

The authors declare that the research was conducted in the absence of any commercial or financial relationships that could be construed as a potential conflict of interest.
